# Inhibition of Acetylcholinesterase and Amyloid-β Aggregation by Piceatannol and Analogs: Assessing In Vitro and In Vivo Impact on a Murine Model of Scopolamine-Induced Memory Impairment

**DOI:** 10.3390/antiox12071362

**Published:** 2023-06-29

**Authors:** Yi-Yan Sie, Liang-Chieh Chen, Cai-Jhen Li, Yu-Hsiang Yuan, Sheng-Hung Hsiao, Mei-Hsien Lee, Ching-Chiung Wang, Wen-Chi Hou

**Affiliations:** 1 Ph.D. Program in Clinical Drug Development of Herbal Medicine, College of Pharmacy, Taipei Medical University, Taipei 110, Taiwan; d339110003@tmu.edu.tw; 2 Department of Pharmacology and Pharmaceutical Sciences, School of Pharmacy, University of Southern California, Los Angeles, CA 90089, USA; lchen895@usc.edu; 3 Graduate Institute of Cancer Biology and Drug Discovery, College of Medical Science and Technology, Taipei Medical University, Taipei 110, Taiwan; 4 Graduate Institute of Pharmacognosy, Taipei Medical University, Taipei 110, Taiwanlmh@tmu.edu.tw (M.-H.L.); 5 School of Pharmacy, Taipei Medical University, Taipei 110, Taiwan; 6 Traditional Herbal Medicine Research Center, Taipei Medical University Hospital, Taipei 110, Taiwan

**Keywords:** acetylcholinesterase, amyloid-β peptide, hydroxylated stilbenes, neurite outgrowth, passive avoidance test, piceatannol (PIC), scopolamine

## Abstract

Currently, no drug is effective in delaying the cognitive impairment of Alzheimer’s disease, which ranks as one of the top 10 causes of death worldwide. Hydroxylated stilbenes are active compounds that exist in fruit and herbal plants. Piceatannol (PIC) and gnetol (GNT), which have one extra hydroxyl group in comparison to resveratrol (RSV), and rhapontigenin (RHA) and isorhapontigenin (isoRHA), which were metabolized from PIC in vivo and contain the same number of hydroxyl groups as RSV, were evaluated for their effects on Alzheimer’s disease-associated factors in vitro and in animal experiments. Among the five hydroxylated stilbenes, PIC was shown to be the most active in DPPH radical scavenging and in inhibitory activities against acetylcholinesterase and amyloid-β peptide aggregations, with concentrations for half-maximal inhibitions of 40.2, 271.74, and 0.48 μM. The different interactions of the five hydroxylated stilbenes with acetylcholinesterase or amyloid-β were obtained by molecular docking. The scopolamine-induced ICR mice fed with PIC (50 mg/kg) showed an improved learning behavior in the passive avoidance tests and had significant differences (*p* < 0.05) compared with those in the control group. The RHA and isoRHA at 10 μM were proven to stimulate neurite outgrowths in the SH-SY5Y cell models. These results reveal that nutraceuticals or functional foods containing PIC have the potential for use in the treatment of neurodegenerative disorders.

## 1. Introduction

Alzheimer’s disease (AD), the main dementia type accounting for over 70% of the entire dementia population [1], clinically features a gradual and progressive decline in memory and executive function (such as short-term memory dysfunctions in organizing and expressing thoughts), the loss of the ability to retain new information, the continuous repetition of statements, failure to remember the names of familiar persons, difficulty in handling complicated tasks, dysfunctions in spatial recognition, and finally, the loss of the ability to participate in general activities [2,3]. AD pathology is characterized by synaptic failures, insoluble amyloid fibrils formed by amyloid-β (Aβ) peptides, and neurofibrillary tangles of aggregates of hyper-phosphorylated Tau proteins [4]. There are two main theories associated with the mechanisms of AD: one is the cholinergic hypothesis [5], and the other is the amyloid cascade hypothesis [6]. Acetylcholine (ACh), which is synthesized by choline acetyltransferase, is a neurotransmitter that is responsible for the establishment and maintenance of memory as the decider between the encoding and retrieval modes in memory processing [7]. Researchers have found that deficient cholinergic neurons, commonly found in the elderly and most Alzheimer’s patients, promote cognitive decline, which is a typical symptom of the disease [8,9]. The cholinergic hypothesis is based on evidence of low choline acetyltransferase activity and low levels of ACh in mild Alzheimer’s patients. The low levels of ACh are expected to enhance long-term depression instead of long-term potentiation and will result in cognitive dysfunction and dementia. During neurotransmission, ACh is released from the presynaptic neuron to bind to and activate the membrane receptor in the postsynaptic neuron, which is then deactivated and hydrolyzed by acetylcholinesterase (AChE) before terminating the nerve impulses [10]. Therefore, therapeutic AChE inhibitors such as donepezil, rivastigmine, and galactamine are developed based on the cholinergic hypothesis and are currently used for AD treatments, which have shown short-term improvements in progressive cognitive decline [11,12]. The amyloid cascade hypothesis has been the mainstream of AD pathological theory in the last 30 years [6], and its key role is the neurotoxicity of different forms of Aβ peptide aggregates, including Aβ peptide oligomers, protofibrils, fibrils, and plaques. The Aβ peptide is produced by beta-site amyloid precursor protein cleaving enzyme 1 (BACE-1) and γ-secretase in the sequential hydrolysis of the β-amyloid precursor protein and residual C-terminal 99 amino acid fragments [13]. Researchers have developed small molecules to inhibit BACE-1 to reduce Aβ peptide levels. Specific macromolecular antibodies have also been exploited to scavenge Aβ peptide monomers, oligomers, fibrils, and plaques. These approaches have proven to be successful in disease-oriented animal models; however, there has been no significant evidence of cognitive behavioral improvement [14]. Until now, only one monoclonal antibody, lecanemab, has been shown to cause a 25% less decline in measures of cognition and function as compared to a placebo [15]; after an 18-month phase-3 trial, it received an accelerated approval by the US FDA for early Alzheimer’s treatments. The pathogenesis of AD cannot be fully explained by a single hypothesis as it might involve protein abnormalities, gradual losses of cholinergic neurons, synaptic failure, metal ion imbalance, oxidative stress, neuro-inflammation, and mitochondrial dysfunction [4,16]. Flavonoids, which exist mainly in fruit, vegetables, and herbal medicines, are a group of naturally occurring polyphenolic compounds with antioxidant and pharmacological activities. Polyphenolic compounds can be used as ingredients in nutraceuticals or functional foods to delay cognitive declines in AD.

**Figure 1:**
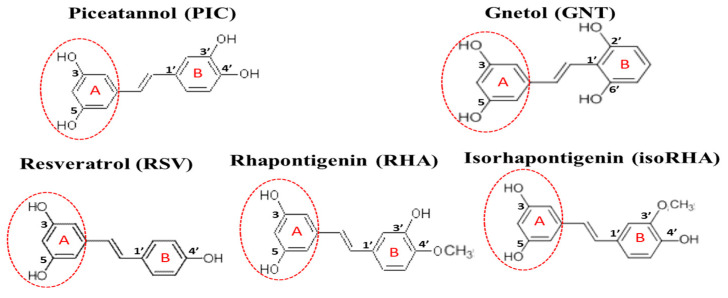
The structures of piceatannol (PIC), gnetol (GNT), resveratrol (RSV), rhapontigenin (RHA), and isorhapontigenin (isoRHA). All compounds contained the same 3,5-dihydroxybenzene group (ring A, dashed red circle).

Piceatannol (PIC, or 3,5,3′,4′-tetrahydroxystilbene, Figure 1) is a naturally occurring polyphenolic compound and exists in fruit and herbal plants, such as *Rheum officinale*, *R. palmatum*, *Rhodomyrtus tomentosa*, passion fruit (*Passiflora edulis*), grape (*Vitis vinifera*), and wild grape (*V. amurensis* or *V. thunbergii*) [17]. In terms of structure, PIC has four hydroxyl groups and one extra hydroxyl group in comparison with resveratrol (RSV, or 3,5,4′-trihydroxystilbene, Figure 1). It can be produced from RSV either through the oxidation of monooxygenase or via the human recombinant protein of cytochrome P450 (CYP)1B1, CYP1A1, and CYP1A2 [17,18] in the presence of nicotinamide adenine dinucleotide phosphate (reduced form, NADPH); another possibility is the total chemical synthesis from different starting materials of vanillin or 3,4-dihydroxybenzaldehyde, with PIC recoveries of 29% and 33%, respectively [19,20]. PIC has been reported to exhibit antitumor, antioxidant, and anti-inflammatory activities [18,21]; to reduce lipid accumulations in 3T3-L1 adipocytes through the inhibition of mitotic clonal expansion [22] or F1F0-ATP synthase [23]; and to reduce blood glucose levels in db/db mice by oral administration (50 mg/kg) for 2–3 weeks [24]. PIC has shown neuroprotective activities against hydrogen peroxide-induced apoptosis in nerve growth factor-treated PC12 cells, with its effect being better than that of RSV [25], and it has also been shown to reduce Aβ-induced neuronal cell death [26,27,28].

The in vivo metabolization of PIC has been proposed, not only via the phase II metabolism of glucuronidation and sulfation but also by catechol-*O*-methyltransferase to generate two metabolites, rhapontigenin (RHA, 3,5,3′-trihydroxy-4′-methoxystilbene, Figure 1) and isorhapontigenin (isoRHA, 3,5,4′-trihydroxy-3′-methoxystilbene, Figure 1) [29]. The structures of RHA and isoRHA contain the same three hydroxyl groups and one extra methoxyl group as compared to that of RSV, and both are reported to exhibit antitumor, antioxidant, and anti-inflammatory activities [30,31,32]. Gnetol (GNT, 3,5,2′,6′-tetrahydroxystilbene, Figure 1) is a naturally occurring polyphenolic compound as an active component of *Gnetum ula* or *G. montanum*, traditionally used for arthritis and asthma treatments, and its structure shows the same four hydroxyl groups as those found in PIC, with two identical positions and two different ones. GNT has been reported to exhibit COX-1 and COX-2 inhibitory activity as well as anti-proliferative activities against cancer cell lines HCT-116 colorectal carcinoma, HepG2 hepatocellular carcinoma, and PC-3 prostate adenocarcinoma [33]. In this study, PIC and its analogs GNT, RSV, RHA, and isoRHA were evaluated in vitro for their effects on Alzheimer’s disease-associated factors, including the DPPH radical scavenging activities, inhibitory activities against AChE, and Aβ peptide (1–42) aggregations and concentrations for 50% inhibitions (IC_50_) of each test compound; the inhibition modes were confirmed by molecular dockings. The scopolamine-induced amnesiac ICR mice with cognitive dysfunction [34,35,36] were treated with PIC to evaluate proof-of-concept improvements in learning behaviors by passive avoidance tests. The SH-SY5Y neuroblastoma cells were used as models to evaluate the neuroprotection conferred by PIC and its analogs against Aβ-induced cell deaths and stimulatory activities on neurite outgrowths. Our results revealed that PIC is a potential ingredient in the development of nutraceuticals or functional foods or can serve as a lead compound for the treatment of an unmet medical need in neurodegenerative disorders.

## 2. Materials and Methods

### 2.1. Chemical and Reagents

PIC, GNT, RSV, RHA, and isoRHA were purchased from Tokyo Chemical Industry Co. (Chuo-ku, Tokyo, Japan) with purity higher than 95%. The acetylthiocholine iodide, Aβ peptide (25–35), dimethylsulfoxide (DMSO), 3-(4,5-dimethylthiazol-2-yl)-2,5-diphenyl tetrazolium bromide (MTT), 2,2-diphenyl-1-picrylhydrazylhydrate (DPPH), 5′,5′-dithiobis(2-nitrobenzoic acid) [DTNB], 1,1,1,3,3,3-hexafluoroisopropanol (HFIP), phosphate-buffered saline (PBS), 0.01% poly-L-lysine solution, scopolamine hydrobromide, and thioflavin T (ThT) were purchased from Sigma Chemical Co. (St. Louis, MO, USA). The 6-hydroxy-2,5,7,8-tetramethylchroman-2-carboxylic acid (Trolox) was purchased from Calbiochem Co. (San Diego, CA, USA). The Aβ peptide (1–42) was synthesized and purchased from Kelowna International Scientific Inc. (New Taipei, Taiwan) with purity higher than 95%. The BCA protein assay kit and the Molecular Probes™ Neurite Outgrowth Staining Kit (A-15001) were purchased from ThermoFisher Scientific Inc. (Rockford, IL, USA). The recombinant human AChE (specific activity of 500 nmol/min/μg) was purchased from R&D Systems Inc. (Minneapolis, MN, USA). Dulbecco’s modified eagle medium (DMEM), fetal bovine serum (FBS), DMEM, and Ham’s F-12 (1:1) mixture medium (DMEM/F-12 medium) were purchased from Gibco BRL Life Technologies (Grand Island, NY, USA).

### 2.2. DPPH Radical Scavenging Activities

DPPH radical scavenging activity was assayed following a previous report [37], with some modifications. A total of 50 μL of PIC and its four analogs at different concentrations (final concentrations of 5, 10, 20, 50, and 100 μM in DMSO) were added into a 96-well plate, and then 150 μL of 120 μM DPPH in methanol was added and reacted at 37 °C under light protection for 20 min. The decrease in absorbance at 517 nm was measured and expressed as △A515 nm. DMSO was used instead of a sample solution in the blank group. Trolox was used as the positive control (the final concentrations were 7.5, 15, 30, 60, 90, and 120 μM in DMSO). The scavenging activity of the DPPH radical (%) was calculated with the following equation: (△A515_blank_ − △A515_sample_) ÷ △A515_blank_ × 100%. The IC_50_ of the DPPH radical scavenging activity of PIC and its analogs or that of Trolox was calculated from each linear equation by three test concentrations and their radical scavenging activities. For Trolox, 30, 60, and 90 μM were used; for PIC, 10, 20, and 50 μM were used; for GNT, RSV, RHA, and isoRHA, 20, 50, and 100 μM were used.

### 2.3. Effects of Piceatannol and Its Four Analogs on AChE Inhibitory Activities In Vitro

Commercially synthesized acetylthiocholine iodide was hydrolyzed using AChE to release thiocholine, which was coupled with DTNB to generate the yellowish 5-thio-2-nitrobenzoate for in vitro AChE activity assays [34,35,36]. For the AChE inhibitory activity assay, each analog was dissolved in the DMSO, and final concentrations of 100, 200, and 500 μM were used for comparisons. An equal volume of DMSO instead of the sample solution was used in the blank group and expressed as 100% AChE activity. The absorbance changes were recorded at 405 nm for 10 min. AChE inhibition (%) was calculated as follows: [(A405_blank_ − A405_sample_)/(A405_blank_)] × 100%. The IC_50_ of the AChE inhibitory activity of PIC and its analogs was calculated from each linear equation with the use of three test concentrations (100, 200, and 500 μM) and their inhibitory activities.

### 2.4. Effects of Piceatannol and Its Four Analogs on Aβ Peptide Aggregations In Vitro

The effects of PIC and its four analogs on the inhibition of Aβ peptide (1–42) self-aggregations were monitored using fluorescent changes in ThT-bound Aβ fibrils [38]. To prepare the 10 μM Aβ peptide working solution, commercial Aβ peptide was first weighted and dissolved in HFIP to 1 mM. Next, the solvent was evaporated in a hood and then re-dissolved using 100 μL of 60 mM NaOH. It was then diluted to 10 μM working solutions with 50 mM phosphate buffer (pH 7.4, containing 150 mM sodium chloride and 1 mM EDTA), following previous reports [31,32,33]. The Aβ peptide (1–42) working solution was mixed with PIC (0.1, 0.25, 0.5, 1 μM in DMSO) or GNT, RSV, RHA, and isoRHA (1, 5, 10, and 20 μM in DMSO), and 10 μM ThT solution was added, mixed, and then gently shaken at 37 °C for 24 h. DMSO instead of the sample solution was used as the control. The 50 mM phosphate buffer was used instead of the Aβ working solution as the sample blank. The Ex_440nm_/Em_486nm_ was recorded at 0 h and 24 h, and changes in fluorescence (△E) over a 24 h period were calculated; the inhibition against Aβ aggregation (%) was calculated as [(△E_control_ − △E_sample_)/(△E_control_)] × 100%. The IC_50_ of the inhibition against the Aβ aggregation of PIC and its analogs was calculated from each linear equation using three test concentrations and their inhibition capacities. For PIC, 0.1, 0.25, and 0.5 μM were used; for GNT, RSV, RHA, and isoRHA, 1, 5, and 10 μM were used.

### 2.5. Molecular Docking In Silico

Docking analysis was performed using the computational software Discovery Studio (DS), a BIOVIA software product, to analyze the molecular modeling, following a previous report [34,39]. The CDOCKER program in DS was used for the molecular docking of AChE and the Aβ peptide (1–42). The Protein Data Bank (PDB) “http://www.rcsb.org/pdb/ (accessed on 12 September 2021)” was used to find information on the crystal structures of AChE (PDB ID:4EY7) “https://www.rcsb.org/structure/4ey7 (accessed on 12 September 2021)” and the Aβ peptide (1–42) (PDB ID:1Z0Q) “https://www.rcsb.org/structure/1z0q (accessed on 12 September 2021)” [40]. The protein structures were prepared using the automatic ligand preparation function in the DS Macromolecules Tools. The AChE binding site was defined as 10 Å from the co-crystallized ligand. The Aβ peptide (1–42) binding site was detected automatically with a 10 Å radius sphere. The docking compounds were prepared using the ligand preparation function in the DS Small Molecules Tools. Other parameters were used in the default parameters.

### 2.6. Effects of Piceatannol and Its Analogs on Aβ Peptide (25–35)-Induced Cell Deaths and Neurite Outgrowths in SH-SY5Y Cell Models

The human SH-SY5Y neuroblastoma cell lines (CRL-2266, Lot No. 58979771) were purchased from American Type Culture Collection (Manassas, VA, USA) and used as models to investigate PIC and its analogs for neuroprotective activities against Aβ-induced cell deaths and the stimulation of neurite outgrowths. The SH-SY5Y cells were grown in DMEM/F-12 medium supplemented with 10% FBS at 37 °C under a humidified atmosphere and 5% CO_2_ [41]. To study the neuroprotective activities against Aβ peptide (25–35)-induced cell deaths, a previous method [42] was modified as follows. The SH-SY5Y cells (10,000 cells/well) were seeded and cultured in a DMEM/F-12 medium containing 10% FBS for 24 h at 37 °C under a humidified atmosphere and 5% CO_2_. After being removed from the culture medium, each PIC, GNT, RSV, RHA, and isoRHA (at a final concentration of 1, 2, and 10 μM in the 0.1%DMSO) or 0.1% DMSO (the control) was added to the medium for a 24 h culture. The medium was then removed, and 40 μg/mL Aβ peptide (25–35) (dissolved in PBS) or an equal aliquot of PBS was added to the medium for another 24 h culture. The MTT was used to evaluate cell viability, and the absorbance at 570 nm was determined [43]. The DMSO used instead of the sample solution was blank (recognized as 100%), and each treatment was expressed as relative cell viability (%). To study the stimulation of neurite outgrowth, the SH-SY5Y cells (5000 cells/well) were seeded on a 96-well plate pre-coated with poly-L-lysine and then cultured in the DMEM/F-12 medium containing 10% FBS overnight at 37 °C under a humidified atmosphere and 5% CO_2_. After being removed from the culture medium, each PIC, GNT, RHA, and isoRHA (at a final concentration of 10 μM in the 0.1%DMSO) or 0.1% DMSO (the control) was added to the DMEM/F-12 medium containing 10% FBS and refreshed every three days. On day 3 and day 6, each of the culture plates was photographed using an inverted microscope (ECLIPSE TS100, Nikon Instruments Inc., Tokyo, Japan). The culture medium was removed and washed with PBS, and the cultured cells in each well were fixed with 4% paraformaldehyde. To measure cell viability and quantify the neurite outgrowths of the treated SH-SY5Y in a 96-well plate, the neurite outgrowth staining kit (a two-color fluorescence assay kit) was used, following a previous report [44] or the protocol in the manufacturer’s manual (publication number: MAN0007224), which included a cell membrane-stained fluorescent dye (orange-red fluorescence), a cell viability indicator (green fluorescence), and a reagent for background masking. The fluorescence ratios of Ex555nm/Em565nm and Ex495nm/Em515nm were determined for the neurite outgrowths and the cell viabilities, respectively. The value for the control was recognized as 100%, and each treatment was expressed relative to the control.

### 2.7. Effects of Piceatannol Pre-Treatments on Cognitive Dysfunctions in Scopolamine-Induced Amnesiac ICR Mice

The scopolamine-induced cognitive dysfunctions of amnesiac ICR mice were used to evaluate the effects of PIC pre-treatments on the improvement of learning and memory functions, following previous reports [34,35,36]. The animal experimental protocols were reviewed and approved by the Institutional Animal Care and Use Committee, Taipei Medical University (No. LAC-2021-0488). After a two-week acclimation, the mice were randomly divided into four groups (N = 5 for each group). In the first stage, the mice in the PIC group were orally pre-treated with PIC (50 mg/kg) using a feeding tube, once a day from day 1 to day 7; the mice in the blank, control, and donepezil groups were treated with the same volume of distilled water from day 1 to day 7. In the second stage, on day 8 and day 9, the mice in the PIC group and the donepezil group were orally treated once with PIC (50 mg/kg) and donepezil (positive control, 5 mg/kg), respectively, using a feeding tube; the mice in the blank and the control groups were treated with the same volume of distilled water. After 30 min, each mouse was injected intraperitoneally with scopolamine (1 mg/kg, dissolved in PBS), and the mice in the blank group were each injected with an equal volume of PBS instead of the scopolamine solution. Again, 30 min after the scopolamine or PBS injection, a learning and memory evaluation was performed on each mouse with the use of a 2-day passive avoidance test. All the mice were sacrificed at the end of the animal experiments, and the brain tissues of the mice in each group were isolated, weighted, and stored at −80 °C for further use.

### 2.8. Learning and Memory Functions in Scopolamine-Induced Amnesiac ICR Mice

A 2-day passive avoidance test, which included an acquisition trial on the first day and a retention trial on the second day, was used to evaluate the learning and memory functions of the scopolamine-induced cognitive dysfunctions of amnesiac ICR mice after PIC and donepezil treatments, following previous reports [34,35,36]. A shuttle chamber (AccuScan Instruments Inc., Columbus, OH, USA) with a light box and a dark box separated by a sliding door was used. On day 8, after acclimation around the shuttle chamber for 30 min, each scopolamine-injected or PBS-injected mouse was placed in the light box for the acquisition trial. Once the mouse entered the dark box, the step-through latency (s) was recorded, and the sliding door was closed at the same time. Immediately after, the mouse was administered an electric foot shock (0.3 mA for 3 s) via the wired metal floor and then sent back to the cage. On day 9, after sample oral administration and scopolamine injection, the same protocol was performed for the retention trial, except for the electric foot shock in the dark box. The step-through latency (period of time that each mouse stayed in the light box) was recorded again. The AChE activities in the mouse brain tissue extract were assayed following the method in Section 2.3, with some modifications. The protein content of the whole brain extracts from the mice was quantified using the BCA protein kit, with bovine serum albumin as the standard protein. To determine the DTNB-coupling products of the non-AChE-generated thiocholine in the assay system (as the sample blank in the brain extracts), donepezil (final concentration of 100 nM) was premixed with each diluted brain extract for 30 min. The AChE activity of each brain extract was calculated as [A405_sample_ − A405_sample blank_]/μg protein, and AChE activity in the blank group was recognized as 100%.

### 2.9. Statistical Analyses

The data were expressed as mean ± SD of three independent experiments, and the step-through latency in the passive avoidance test was expressed as mean ± SE. The Aβ-induced cell deaths or neurite outgrowth in the SH-Y5Y cells between the control and each sample treatment or between the control and the blank were analyzed using Student’s *t*-test, and any difference in comparison with the control group was considered statistically significant when *p* < 0.05 *, or *p* < 0.01 **, or *p* < 0.001 ***. Multiple group comparisons among animal experiments were performed using the one-way analysis of variance (ANOVA) and the post hoc Tukey’s test, and the different lowercase (for the acquisition trial) or uppercase letters (for the retention trial or the AChE activity in the brain extracts) in each bar were considered significantly different (*p* < 0.05). Statistical analysis was performed using the GraphPad Prism 6.0 software (San Diego, CA, USA).

## 3. Results

### 3.1. Effects of PIC and Its Analogs on DPPH Radical Scavenging Activity, AChE Inhibitory Activities, Inhibitions against Aβ Peptide (1–42) Aggregations, and Neuroprotection against Aβ-Induced Cell Deaths

**Figure 2:**
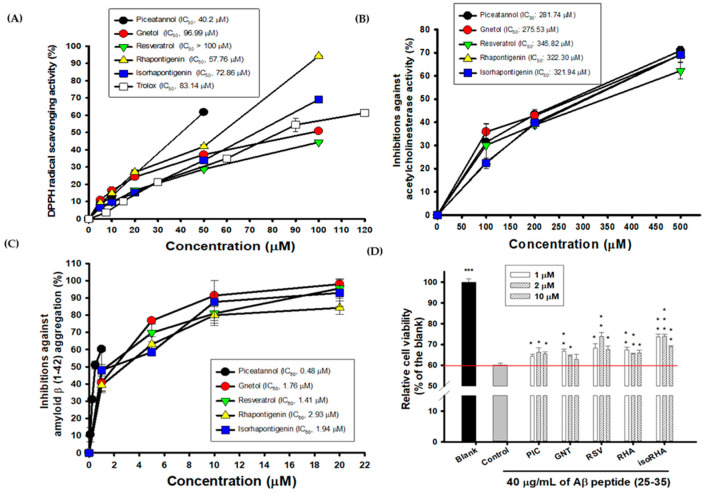
Biological activities of PIC and its analogs. (**A**) DPPH radical scavenging activity; (**B**) AChE inhibitory activities; (**C**) inhibitions against Aβ peptide (1–42) aggregations; (**D**) neuroprotection against Aβ peptide (25–35)-induced cell deaths. IC_50_ represented the concentrations for 50% inhibition in the present study. The Aβ-induced cell deaths in SH-Y5Y cells between the control and the blank or the control and each sample treatment were analyzed using Student’s *t*-test, and any difference in comparison with the control group was considered statistically significant when *p* < 0.05 *, or *p* < 0.01 **, or *p* < 0.001 ***.

The hydroxylated stilbenes of PIC and its analogs, GNT, RSV, RHA, and isoRHA (Figure 1), were used to evaluate in vitro biological activities. All compounds contained the same 3,5-dihydroxybenzene group (ring A, dashed red circle). Figure 2A shows the DPPH radical scavenging activities. It was found that PIC and its analogs showed dose-dependent DPPH radical scavenging activities, and the IC_50_ of the DPPH radical scavenging activities of PIC, GNT, RSV, RHA, and isoRHA were, respectively calculated to be 40.2 μM, 96.99 μM, higher than 100 μM, 57.76 μM, and 72.86 μM in this study’s assays. The IC_50_ of the positive control, Trolox, was 83.14 μM. The DPPH scavenging capacity of PIC was 2.5-fold and 2.07-fold higher than that of RSV and Trolox, respectively. The scavenging order of the DPPH radical was PIC > RHA > isoRHA > Trolox > GNT > RSV. Figure 2B shows the in vitro anti-AChE activities among the five hydroxylated stilbenes. It was found that PIC and its analogs, GNT, RSV, RHA, and isoRHA, showed dose-dependent AChE inhibitory activities, and the IC_50_ of the anti-AChE activities of PIC, GNT, RSV, RHA, and isoRHA were calculated to be 281.74 μM, 275.53 μM, 345.82 μM, 322.30 μM, and 321.94 μM, respectively, in this study’s assays, with the AChE inhibitory capacity of PIC being 1.23-fold higher than that of RSV. The inhibitory order against AChE in the present results was GNT ≅ PIC > isoRHA ≅ RHA > RSV. Figure 2C shows the in vitro anti-Aβ peptide (1–42) aggregations among five hydroxylated stilbenes. It was found that PIC and its analogs showed dose-dependent inhibitions against Aβ peptide (1–42) aggregations. The inhibitory order against the Aβ peptide aggregations was PIC > RSV > GNT > isoRHA > RHA. The IC_50_ of the anti-Aβ peptide aggregations of PIC, GNT, RSV, RHA, and isoRHA were calculated to be 0.48 μM, 1.76 μM, 1.41 μM, 2.93 μM, and 1.94 μM, respectively, in the present assays, and the anti-Aβ aggregations capacity of PIC was 2.94-fold higher than that of RSV. Figure 2D shows the neuroprotective activities against the Aβ peptide (25–35)-induced cell deaths in SH-SY5Y cell models. The treatment of 40 μg/mL of the Aβ peptide (25–35) showed a reduction in cell viability from 100% to about 60% (control). With each compound having insignificant cytotoxicity (Supplementary Figure S1), the pre-treatment of PIC, GNT, RSV, RHA, and isoRHA (1, 2, and 10 μM) for 24 h showed that the neuroprotection elevated cell viabilities and produced significant differences as compared to the control (*p* < 0.05 *, 0.01 **, 0.001 ***).

### 3.2. Molecular Dockings of PIC and Its Analogs on AChE Inhibitions

**Figure 3:**
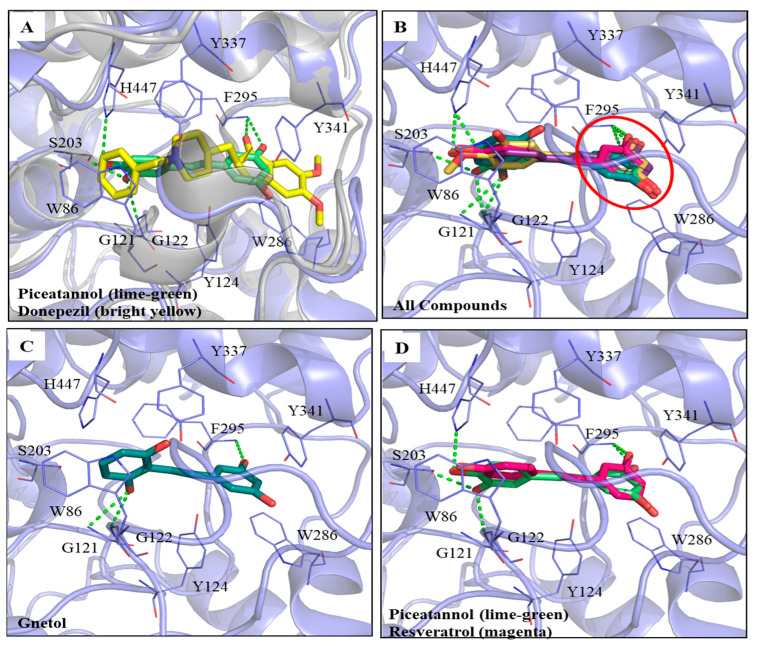
Interactions analysis between AChE (PDB ID: 4EY7) and PIC or its analogs. (**A**) Superimpose docking poses of donepezil (bright yellow) and PIC in active site regions of AChE; (**B**) Docking poses of gnetol (blue-green), piceatannol (lime-green), isorhapontigenin (purple), rhapontigenin (yellow), and resveratrol (magenta) in the active site of AChE (light blue). The 3,5-dihydroxybenzene group (ring A, red circle); (**C**) The docking pose of GNT in active site regions of AChE; (**D**) Superimposed docking poses of RSV and PIC in AChE active site. Hydrogen bonds were represented as green dash lines. Residues were labeled as shown. The figures were created using Pymol.

The computational software Discovery Studio (DS) was used to analyze the simulated interactions between the compounds and key amino acid residues in the proteins. The docking protocol was validated by re-docking the co-crystallized ligand (donepezil) into the binding site. The re-docking result displayed a similar conformation to that of the co-crystallized ligand (Supplementary Figure S2), with a root mean square deviation (RMSD) value of 1.6494 Å, suggesting that a reliable docking protocol had been used. Figure 3A shows the superimposed docking results of PIC (lime-green) and donepezil (bright yellow). The PIC occupied the active site of AChE. Figure 3B shows the superimposed docking poses of the five compounds, GNT (blue-green), isoRHA (purple), PIC (lime-green), RHA (yellow), and RSV (magenta), in the active site of AChE (light blue). It was found that all these hydroxylated stilbenes occupied the active site of AChE and displayed similar conformations (Figure 3B). The 3-hydroxyl group of ring A (red circle) formed a hydrogen bond with residue Phe295 (F295) in the AChE. Additionally, the aromatic ring A also generated a π–π stacked interaction with residue Trp286 (W286) in the AChE. These interactions stabilized these compounds with the peripheral anionic site (PAS) of AChE. The docking poses of the first two (GNT and PIC) and the last (RSV) potent AChE inhibitions among these five hydroxylated stilbenes are separately described as follows. GNT is shown to be the most potent AChE inhibitor of these hydroxylated stilbenes in Figure 2B. In the GNT–AChE complex (Figure 3C), GNT formed hydrogen bonds not only with F295 but also with residues Gly121 (G121) and G122 near the catalytic anion site (CAS) of AChE. In addition to polar contacts, GNT also formed hydrophobic interactions with AChE, including a π–π stacked interaction with residue W286 and three π–π T-shaped interactions with residues Tyr124 (Y124), Y337, and His447 (H447). PIC showed similar AChE inhibitory activity to that of GNT in Figure 2B. Besides the interactions formed by ring A that are similar to those formed by other hydroxylated stilbenes in the present study, the 3′-hydroxyl group of PIC could form a hydrogen bond with residue G122 near the CAS of AChE (Figure 3A). The 3′,4′-dihydroxyl group of PIC extended to the esteratic site (ES) of AChE and formed two hydrogen bonds with residues Ser203(S203) and H447. Moreover, PIC also showed π–π T-shaped interactions with residues Y124 and H447, similar to those of GNT, which resulted in the similarity of the AChE inhibitions of GNT and PIC in the present study. The docking pose of RSV was compared with that of PIC (Figure 3D). The structural difference between RSV and PIC was only in the 3′-position. RSV showed the same interactions in ring A and formed one hydrogen bond with residue F295. Furthermore, the aromatic ring A also generated a π–π stacked interaction with residue W286 in the AChE; however, no hydrogen bond was formed in the CAS or ES region of AChE, which demonstrated that fewer interactions result in differences in AChE inhibitory activity. The CDOCKER interaction energy results are summarized in Supplementary Table S1. The table shows the lowest interaction energy of the docked PIC–AChE (−36.3392 Kcal/mol) and the highest interaction energy of the docked RSV-AChE (−31.4709 Kcal/mol) from among five hydroxylated stilbenes using the CDOCKER program in DS, which reveals the most stable PIC–AChE complex and the highest AChE inhibitions in the present study.

### 3.3. Molecular Dockings of PIC and Its Analogs on Inhibitions against Aβ Peptide Aggregations

**Figure 4:**
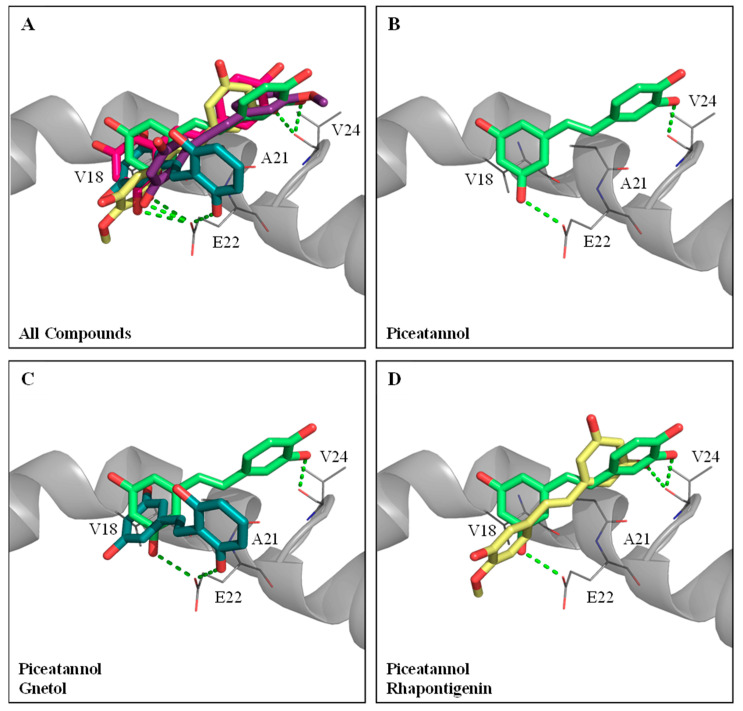
Molecular docking analysis between Aβ peptide (1–42) (PDB ID:1Z0Q) and PIC or its analogs. (**A**) The docking poses of piceatannol (PIC, lime-green), gnetol (GNT, blue-green), resveratrol (RSV, magenta), rhapontigenin (RHA, yellow), and isorhapontigenin (isoRHA, purple) in the same segment of Aβ peptide (1–42) helix (gray). (**B**) The docking pose of PIC in the segment of Aβ peptide (1–42) helix. Superimposed docking poses of (**C**) GNT and PIC, and (**D**) RHA and PIC in a segment of Aβ peptide (1–42) helix. Hydrogen bonds were represented as green dash lines. Residues are labeled as shown. The figures were created using Pymol.

The five hydroxylated stilbenes were docked into the Aβ peptide (1–42) (PDB ID:1Z0Q). All compounds contained the same 3,5-dihydroxybenzene group (ring A) with different substitutes for the hydroxyl group or the methoxyl group in ring B (Figure 1). However, the Aβ peptide (1–42) does not have any known crystalized structure with a co-crystallized ligand. Therefore, the binding site was set to cover the whole protein. It was found that all compounds occupied the same segment of the Aβ peptide (1–42) helix (Figure 4A) with different interactions. The docking poses of GNT (blue-green), PIC (lime-green), isoRHA (purple), RHA (yellow), and RSV (magenta) in the Aβ peptide (1–42) (gray) are shown in the figure. All five compounds occupied and interacted with the same region (amino acid residues 18–24) of the Aβ peptide (1–42). The docking poses of PIC, GNT, and RHA are separately described below.

PIC proved to be the most potent inhibitor against the Aβ peptide (1–42) self-aggregations of these hydroxylated stilbenes, as shown in Figure 2C. The docking pose of PIC (lime-green) is shown in Figure 4B. It was found that PIC formed two hydrogen bonds, in which ring A interacted with residue Glu22 (E22) and ring B interacted with residue Val24 (V24); meanwhile, ring A also formed π–alkyl interactions with residues V18 and Ala21 (A21). The docking pose of GNT (blue-green) is shown in Figure 4C, and the PIC (lime-green) is used for comparison. It was found that ring B (3′-hydroxyl group) of GNT formed one hydrogen bond with residue E22, which was shown to interact with ring A in the PIC. Meanwhile, ring A of GNT also formed π–alkyl interactions with residues V18 and A21, which were similar to those formed by PIC. The docking pose of RHA (yellow) is shown in Figure 4D, with PIC (lime-green) used for comparison. Owing to the steric hindrance of residue V24 with the 4′-methoxyl group (ring B) in RHA (but no steric hindrance of residue V24 with the 3′-methoxyl group in isoRHA), the opposite docking poses were displayed between RHA and PIC. The only 3-hydroxyl group (ring A) of RHA formed one hydrogen bond with residue V24, which interacted with ring B in PIC, and no further interaction of the Aβ peptide (1–42) with RHA was found, which might have produced the lowest inhibition against the Aβ (1–42) aggregations in the present study. The CDOCKER interaction energy results are summarized in Supplementary Table S1. Using the CDOCKER program in DS, it was found that the two lowest interaction energies of the docked PIC-Aβ (1–42) and the docked RSV-Aβ (1–42) from among the five hydroxylated stilbenes were, respectively, −19.3083 Kcal/mol and −17.0259 Kcal/mol, which meant that the most stable complexes are those of PIC–Aβ (1–42) and RSV–Aβ (1–42) in the present study.

### 3.4. Effects of Piceatannol and Its Analogs on Neurite Outgrowths in SH-SY5Y Cell Models

**Figure 5:**
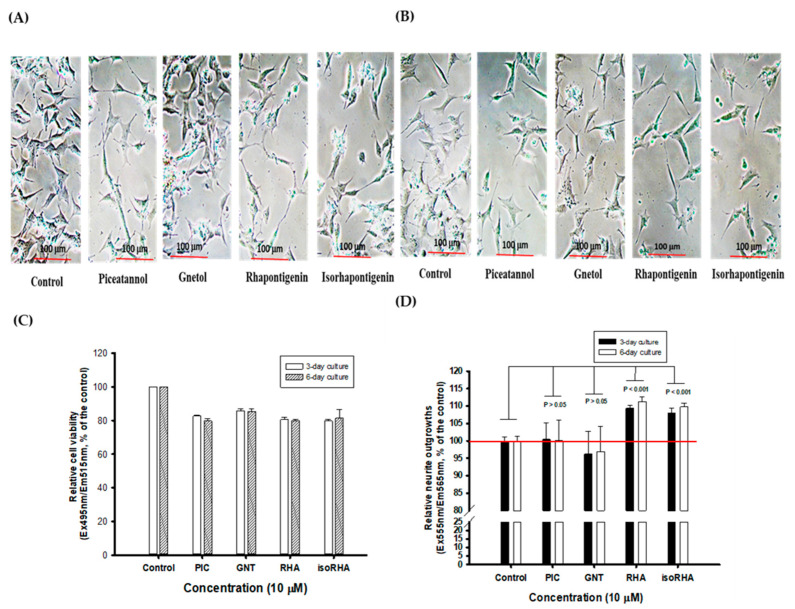
The photographs of (**A**) 3-day and (**B**) 6-day cultures without (control) or with treatments of 10 μM of piceatannol (PIC), gnetol (GNT), rhapontigenin (RHA), and isorhapontigenin (isoRHA) on neurite outgrowths in SH-SY5Y cells by the inverted microscope (200-fold magnifications). A two-color fluorescence assay kit was used, and (**C**) the green fluorescence (Ex495nm/Em515nm) was used to assay cell viability; and (**D**) the orange-red fluorescence (Ex555nm/Em565nm) with background masking was used to determine the effects on stimulated neurite outgrowths. The ratio of fluorescence in the control was recognized as 100%, and each treatment was expressed relative to the control (%). Two group comparisons were analyzed using Student’s *t*-test, and any difference in comparison with the control group was considered statistically significant when *p* < 0.05, 0.01, or 0.001.

Figure 5A and 5B, respectively, show photographs of the 3-day and 6-day cultures without (control) or with 10 μM treatments of PIC, GNT, RHA, and isoRHA on the neurite outgrowths in SH-SY5Y cells, which were taken using an inverted microscope (200-fold magnification). To determine the stimulated neurite outgrowths in SH-SY5Y cells after the 3-day and 6-day treatments by hydroxylated stilbenes, a two-color fluorescence assay kit was used in the present study. Figure 5C shows the cell viability of SH-SY5Y cells after the 3-day or 6-day treatments, as detected by green fluorescence. It was found that the 10 μM PIC, GNT, RHA, and isoRHA treatments, after either 3 days or 6 days, maintained 80% cell viability relative to the control. Figure 5D shows the determinations of neurite outgrowths in SH-SY5Y cells after the 3-day or 6-day treatments, as detected by orange-red fluorescence with background masking. It was found that RHA and isoRHA, but not PIC nor GNT, showed stimulated activities toward neurite outgrowths after the 3-day or 6-day treatments and had a significant difference (*p* < 0.001) as compared to the control maintained 80% cell viability relative to the control. Figure 5D shows the determinations of neurite outgrowths in SH-SY5Y cells after the 3-day or 6-day treatments, as detected by orange-red fluorescence with background masking. It was found that RHA and isoRHA, but not PIC nor GNT, showed stimulated activities toward neurite outgrowths after the 3-day or 6-day treatments and had a significant difference (*p* < 0.001) as compared to the control.

### 3.5. Effects of Piceatannol Pre-Treatments on Improvements in the Cognitive Dysfunctions of Scopolamine-Induced Amnesiac ICR Mice

**Figure 6:**
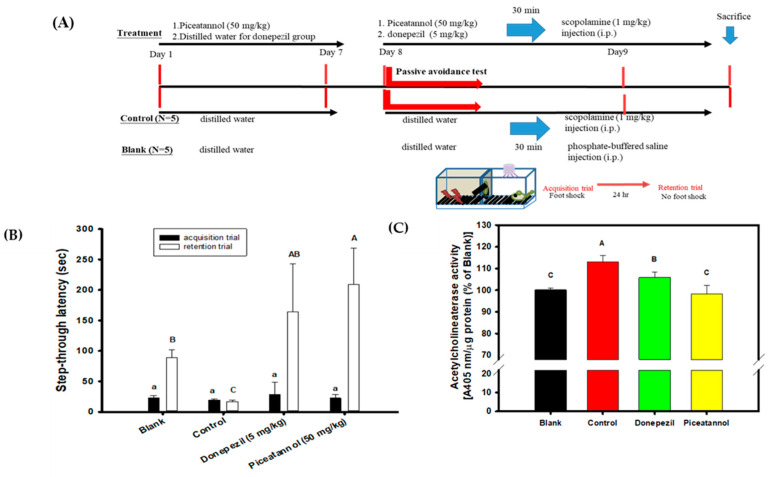
Effects of piceatannol pre-treatments on improvements of cognitive dysfunctions in scopolamine-induced amnesiac ICR mice. (**A**) The preventive protocol of PIC pre-treatment once a day (50 mg/kg) for 7 days before a learning behavior evaluation and donepezil (5 mg/kg) was used as the positive control. (**B**) The step-through latency (s) of mouse in a two-day trial of the passive avoidance test, the first day was the acquisition trial and the second day was the retention trial. (**C**) The AChE activity in the brain extracts of the blank and the treated groups. Multiple group comparisons were performed using one-way analysis of variance (ANOVA) and the post hoc Tukey’s test, and the different lowercase alphabet letters (for the acquisition trial) or uppercase alphabet letters (for the retention trial or the AChE activity in the brain extracts) in each bar were considered significantly different (*p* < 0.05).

PIC showed biological activities in vitro that had the highest association with the delayed onset of AD among the five hydroxylated stilbenes in the present study. These included AChE inhibitory activities, anti-Aβ peptide (1–42) self-aggregation, and free radical scavenging activities. Therefore, PIC was administered orally in the animal experiment to evaluate the improvements in the cognitive dysfunctions of scopolamine-induced amnesiac ICR mice. Figure 6A shows the preventive protocol of PIC pre-treatment, with administration carried out once a day (50 mg/kg) for 7 days before a learning behavior evaluation (a passive avoidance test). This included a two-day trial: the acquisition trial was performed on the first day and the retention trial on the second day. Donepezil (5 mg/kg) was used as the positive control. Figure 6B shows the results of the step-through latency (s) of mice from a two-day trial using the passive avoidance test. The step-through latency (s) on the first day (the acquisition trial) showed no significant difference among the groups (the same lower alphabetic letter, *p* > 0.05), as determined with the use of one-way ANOVA and the post hoc Tukey’s test. The step-through latency (s) of the blank, the donepezil group, or the PIC-treated group on the second day (the retention trial) showed a longer staying time and had significant differences compared to that of the control (uppercase alphabetic letter, *p* < 0.05), as determined by one-way ANOVA and the post hoc Tukey’s test. The present results show that PIC pre-treatment (50 mg/kg) once a day for 7 days improved the scopolamine-induced cognitive dysfunctions of amnesiac ICR mice and were comparable to those in the donepezil-treated group. Figure 6C shows the results of mouse AChE activity in the brain extracts of the blank and the treated groups. The mice in the control group showed the highest AChE activity in the brain extracts via the scopolamine injections; however, PIC-treated mice or donepezil-treated mice showed reductions in AChE activities in the brain extracts and showed significant differences compared to those in the control group (uppercase alphabetic letter, *p* < 0.05), as determined by one-way ANOVA and the post hoc Tukey’s test.

## 4. Discussion

This is the first report to demonstrate that daily PIC pre-treatments for 7 days can improve the learning and memory functions of scopolamine-induced amnesiac mice with cognitive dysfunctions, which may have potential benefits by delaying the onset of AD. The hydroxylated stilbene of PIC showed free radical scavenging activities as well as inhibitory activities against AChE, Aβ peptide (1–42) aggregations, and Aβ peptide (25–35)-induced cell deaths in vitro. In addition, a proof-of-concept improvement was observed in the cognitive dysfunctions of ICR mice via the passive avoidance test. The administered doses in animal experiments and the concentrations used in human clinical trials were proposed to be mutually translated via body surface area normalizations by multiplying the *Km* factor (body weight (kg) divided by body surface area (m^2^)) of each human and experimental animal used rather than by multiplying the different body weights of two translated species [45]. Therefore, the human equivalent concentration of the PIC treatment dose (50 mg/kg) for scopolamine-induced ICR mice was calculated to be 4.054 mg/kg, and an adult weighing 60 kg is expected to receive similar beneficial effects by taking about 250 mg of PIC per day. However, further investigations in clinical trials are needed.

Fruit, vegetables, and herbal medicines rich in polyphenols have been reported to exhibit neuroprotective functions. However, the bioavailability, blood–brain barrier, as well as the neuroprotection and neurotoxicity of polyphenols are still largely problematic for future use [46]. High amounts of PIC can be found in the herbal medicine *Polygonum cuspidatum* (25–67 μg/g) [18], in seeds of *Passiflora edulis* (2.2 mg/g of raw seed) [47], and in hairy root cultures of peanut (348.75–550.21 μg/g of dry weight or 4.02–5.4 mg/L of culture medium) [48]. The possible dietary sources of PIC are black tea (14 or 53 μg/g), green tea (14 or 53 μg/g), red grape (24 or 374 ng/g), white grape (43 ng/g), or wine (6.1 or 14 ng/mL) [18]. Besides the intake of tea or grapes, nutraceuticals or functional foods containing PIC could be an alternative route, for which it is necessary to carry out extraction and/or purification procedures that would increase the amounts of PIC to obtain its beneficial effects. The purification protocols need to be established in the near future. It was also reported that the oral administration of RSV (75 mg/kg) to athymic nude mice enabled not only the detection of RSV and RSV-glucuronide in the plasma, liver, and skin after 5 min, but also the detection of PIC in the plasma (5.25 μmol/L), liver (11.50 nmol/g), and skin (2.4 nmol/g) [49]. The oral administration of RSV allowed it to be metabolized rapidly and generate PIC in vivo. The bio-availabilities and half-lives of PIC and RSV following their oral administration to rats were 50.7% and 4.23 h and 29.8% and 1.48 h, respectively [50]. According to the pharmacokinetic analyses of the intragastric administration of PIC or RSV (90, 180, and 360 μmol/kg) to rats, the area under the curve at 0–8 h (AUC_0–8h_) in plasma for intact PIC and RSV were 4.3, 12.3, 20.6 (μmol.h/L) and 2.0, 4.7, 8.6 (μmol.h/L), respectively; after the co-administration of PIC and RSV (each at 180 μmol/kg) to rats, the AUC_0–8h_ in plasma for intact PIC and intact RSV were 8.6 and 4.1 (μmol.h/L), respectively [51]. These pharmacokinetic results in rats reveal that PIC is about two times better than RSV in terms of bioavailability and half-life in rat plasma. It was noted that the AUC_0–8h_ of intact PIC/AUC_0–8h_ of total PIC metabolites was only 16.3% in the co-administration of PIC and RSV (each at 180 μmol/kg), and only 18.6%, 23.9%, and 25.1% in the administration of PIC (90, 180, and 360 μmol/kg) [51]. Parts of the metabolites of PIC in vivo might exhibit pharmacological activities together with the intact PIC to overcome the threshold concentration for the maintenance of biological activities; this will need further investigation. It was suggested that the delivery formulations, such as α-cyclodextrin-PIC or β-cyclodextrin-PIC [52], and PIC-loaded emulsomes [53] could increase the solubility and bioavailability of PIC in vivo.

Until now, no human clinical trial of PIC has been conducted nor made available. RSV is a well-known hydroxylated stilbene with several biological activities [54,55,56]. RSV has been used for treatment in different AD animal models (APP/PS1 transgenic mice, Tg199589 transgenic mice, SAMP8 mice, Aβ-injected SD rats, and ovariectomized rats with chronic galactose injections) and for the treatment of healthy older adults, mild AD patients, or risk factors-associated AD people in clinical trials. The results of these trials showed that RSV exhibits benefits related to the reduction in AD-associated biomarkers and inflammations. RSV has also been proven to improve spatial memory in animal models and to decrease Aβ levels in clinical trials [57,58]. A 52-week human clinical trial of RSV was carried out for mild to moderate AD patients (four stages with 13-week intervals, initially 500 mg once daily up to 13 weeks, and a dose escalation of 1000 mg twice daily from 39 weeks to 52 weeks) [59,60]. These human trial results showed that: RSV could cross the blood–brain barrier (BBB), the used RSV doses were safe and had good tolerance with adverse effects of nausea, diarrhea, and weight loss, the Aβ42 and Aβ40 levels in the cerebrospinal fluid were reduced, and RSV intake could modulate neuro-inflammation and decrease cognitive decline; the side effect was an increase in brain volume. PIC was shown to protect BBB integrity in lipopolysaccharide-induced inflammation and oxidative stress in mouse brain microvascular endothelial cell models [61]. No information on PIC crossing the BBB was found; however, the glycoside of PIC (piceatannol-3′-*O*-β-d-glucopyranoside) was reported to cross the BBB [62], which might be beneficial in the preventive use of PIC as it can delay the learning and memory dysfunctions caused by AD.

PIC performed better than RSV in the present in vitro assay systems, particularly in terms of DPPH radical scavenging activities, AChE inhibitory activities, and anti-Aβ peptide (1–42) self-aggregations. For the DPPH radical scavenging activities, the scavenging order was PIC > RHA > isoRHA > GNT > RSV. PIC has been reviewed to have more radical scavenging activities (DPPH radicals and superoxide radicals) and higher oxygen radical absorbance capacity than RSV [18]. The first two potent DPPH scavengers of PIC and RHA showed the same moieties, those of the 3,5-dihydroxybenzene group (ring A) and the 3′-hydroxyl group in ring B. The isoRHA and RSV had fewer DPPH scavengers, which shared the same moieties, such as those of the 3,5-dihydroxybenzene group (ring A) and the 4′-hydroxyl group in ring B. PIC and GNT exhibited four hydroxyl groups in their structures. RHA, isoRHA, and RSV exhibited three hydroxyl groups in their structures. Therefore, it was proposed that the DPPH radical scavenging activity was not only dependent on the number of hydroxyl groups in the five hydroxylated stilbenes; more importantly, it was dependent on the position of the hydroxyl group, such as the additional hydroxyl group exhibited by PIC in the 3′ position (ring B).

For the AChE inhibitory activities, the inhibitory order was GNT ≅ PIC > isoRHA ≅ RHA > RSV. The molecular docking results (Figure 3) clearly illustrate the AChE inhibitory activities of hydroxylated stilbenes. These show the more stable interactions between the key amino acid residues of AChE and the hydroxylated stilbenes, which resulted in the reduction in AChE activity through the occupation of the active site of AChE. The docking pose of each hydroxylated stilbene used in the present study was based on a previous report on the donepezil–AChE interactions [34], also shown in Figure 3A. The RSAM value of the re-docking result displayed a similar conformation to that of the co-crystallized ligand of 1.6494 Å, suggesting that the docking protocol used in the present study was reliable. The common structure of ring A in the five hydroxylated stilbenes formed a hydrogen bond with residue F295 and generated a π–π stacked interaction with residue W286 in the AChE, all of which existed in the docking pose of the indanone moiety of donepezil–AChE. GNT also additionally formed a π–π stacked interaction with residue Y337, which was found in the docking pose of the piperidine ring of donepezil–AChE [34].

For the anti-Aβ peptide (1–42) self-aggregations, the inhibitory order was PIC > RSV > GNT > isoRHA > RHA. Based on the molecular docking results, all five hydroxylated stilbenes occupied and interacted with amino acid residues 18–24 of the Aβ peptide, which was positioned at the Aβ aggregation core of amino acid residues 16–21 [63]. The 4′-methoxyl group in RHA and the 3′-methoxyl group in isoRHA resulted in different docking poses with the Aβ peptide for the steric hindrance of residue V24. The isoRHA had the same docking pose as that of PIC, and the RHA showed a reversed docking pose in comparison to that of PIC. The β-sheet structures of the Aβ peptides tended to form oligomers, polymers, and insoluble fibrils via hydrophobic and π–π interactions among the aggregation cores [64]. This could be interrupted by the stable binding of hydroxylated stilbenes within the regions, which would render the secondary α-helix of the Aβ peptide susceptible to conformational changes in the β-sheet [39]. It was found that two hydrogen bonding interactions (3-OH interacting with residue E22 and 3′-OH interacting with residue V24) occurred between PIC and the aggregation core region of Aβ rather than only one (3-OH interacting with residue E22) between RSV and the aggregation core region of Aβ, which allowed PIC to form a more stable hybrid with the Aβ peptide than that containing RSV and resulted in higher inhibition against the Aβ self-aggregations in the present study. It has been reported that AChE showed abilities to accelerate Aβ aggregation and amyloid formation via the Aβ–AChE complex that enhance neurotoxicity [65,66]. It was proposed that the development of AD factors associated with “multi-target small molecules” [67] or “multifaceted hybrid agents” [16] seemed better than the “one molecule-one target” used for AD therapy. Furthermore, the multiple targets of PIC exhibited AChE inhibitory activities (Figure 2B), anti-Aβ self-aggregation (Figure 2C), neuroprotection against Aβ(25–35)-induced cell deaths (Figure 2D), and antioxidant and anti-inflammation abilities [18,21], which might be beneficial in delaying the onset of AD.

RHA and isoRHA at 10 μM, but not PIC and GNT, showed stimulatory activity in neurite outgrowths as compared to the control (DMSO in the cultured medium) in SH-SY5Y cell models using fluorescent dyes for cell membrane stains. At the same time, the cell viabilities of the PIC-, GNT-, RHA-, and isoRHA-treated groups were kept at around 80% of the control group. It was calculated that the total neurite lengths in living SH-SY5Y cells after RHA and isoRHA treatments might reach a size that is 1.375-fold that of the control, and the associated mechanisms should be investigated further. The synthetic gentiside-derived compounds [68], and natural curcuminoids of curcumin, demethoxycurcumin, and bisdemethoxycurcumin [69] showed neuritogenic activities in PC12 cells similar to functions of the nerve growth factor. It has been reported that the treatment of rat hippocampal primary neurons with the Aβ peptide (1–42) oligomer resulted in synaptic dysfunction and a loss of dendrite complexity in vitro [70] and that these symptoms had also been found in the brains of patients with early onset AD [71]. A diarylheptanoid compound, 7-(4-hydroxyphenyl)-1-phenyl-4E-hepten-3-one, isolated from the medicinal plant *Alpinia officinarum*, was shown to not only promote neuronal differentiations and neurite outgrowths in neuroblastoma Neuro-2a cell models [72] but also to exhibit neuroprotective activities that elevate cell viabilities and enhance dendritic complexity levels and dendritic branches against Aβ oligomers-induced neurotoxicity in the cultured hippocampal neurons of rat embryos [73].

Through the intravenous administration of PIC (90 μmole/kg or 21.98 mg/kg) to SD rats, PIC was not only metabolized mainly to glucuronidated-PIC (*C*max was 85.5 ± 67.0 μM), but it also generated the RHA (*C*max was 1.10 ± 0.29 μM) and isoRHA (*C*max was 5.08 ± 0.31 μM) catalyzed by COMT; the RHA and isoRHA were further eliminated by glucuronidation metabolism [29]. It was noted that RSV, RHA, and isoRHA in vivo were metabolized only by the phase II pathway (glucuronidation and sulfation) [29]. In the literature, PIC, RHA, and isoRHA have been shown to exhibit antitumor, antioxidant, and anti-inflammatory activities [18,21,30,31,32]. As reported in the literature, therefore, the scopolamine-induced amnesiac mice with cognitive dysfunctions were used to evaluate the proof-of-concept of PIC oral administration in improving learning and memory functions.

Scopolamine, a medication used to prevent motion sickness [74], was tentatively applied to interrupt the effects of acetylcholine to induce impaired memories by competitively binding to muscarinic receptors. This transient dysfunction could then be ameliorated by applying AChE inhibitors. Consequently, scopolamine has been used in AD animal experiments and human clinical trials to develop the efficacy of AChE inhibitors [34,35,36,75,76,77]. Pre-treatments with vitisin A (40 mg/kg), a resveratrol tetramer isolated from *Vitis thunbergii* var. *taiwaniana*, showed improved learning and memory functions via the passive avoidance test in scopolamine-induced amnesiac ICR mice with cognitive impairments [36]. The pre-treatment of the resveratrol dimer of ampelopsin A was delivered via a cannula implant into the third ventricle of the brain (a total of 65 ng/month/head of C57BL/6), and cognitive impairments were then induced in amnesiac ICR mice via the administration of scopolamine [78]. It was found that the one-month pre-treatment of ampelopsin A in mice with cognitive impairment prompted the clear restoration of learning behavior, as verified through the passive avoidance test and novel object recognition. The two-day passive avoidance test involved dealing with long-term emotional memory strengthened by stressful stimuli, and the improved behavior of experimental animals relied on effective communication that was stimulated by the test compounds or drugs applied between the amygdala and the hippocampus [79]. An intraperitoneal injection of lipopolysaccharide (LPS, 0.8 mg/kg) to induce systemic inflammation and impaired cognition in Swiss albino mice was used to evaluate the effect of 6-day PIC treatments (2.5 mg/kg/day) on learning behavior, in addition to the use of the Y maze test and the object recognition test [80]. Although the LPS-induced animal model is not commonly used for AD treatment, it was found that a 6-day PIC treatment [80] could ameliorate not only learning and memory functions, but also reduce Aβ levels in brain extracts of LPS-treated mice. The limitations of the PIC oral administrations in the present study might include the following points. The dose effects of PIC in scopolamine-induced mice were not clear; the metabolites or pharmacokinetic properties of PIC pre-oral administrations could be determined in the brain regions and the plasma; and the APP/PS1 transgenic mice could be used to evaluate the effects of PIC treatments on Aβ amyloid plaques and the learning behaviors.

## 5. Conclusions

PIC showed the best performance in terms of DPPH radical scavenging activities, AChE inhibitory activities, and anti-Aβ (1–42) self-aggregations from among the five hydroxylated stilbenes examined in the present in vitro study. It also exhibited neuroprotection capabilities against Aβ-induced cell deaths, which these stilbenes containing the same 3,5-dihydroxybenzene group in their structures. PIC also exhibited the proof-of-concept improvement in learning and memory functions, as evaluated by the passive avoidance test in scopolamine-induced amnesiac mice with cognitive dysfunctions. The molecular docking results revealed the interactions between PIC and amino acid residues in the active site of AChE, in which the stable PIC–AChE complex might block access to acetylcholine for further hydrolysis. The molecular docking in silico also revealed the interactions between PIC and the aggregation core of the Aβ peptide, in which the stable complex PIC–Aβ (α helix) might prevent the formation of β-sheet conformation, Aβ oligomers, Aβ polymers, and fibrils. Therefore, PIC is a potential ingredient for the development of nutraceuticals and functional foods, or as a lead compound for an unmet medical need in neurodegenerative disorders.

## Data Availability

All figures and data used to support this study are included within this article.

## Supplementary Materials

The following supporting information can be downloaded at: https://www.mdpi.com/article/10.3390/antiox12071362/s1. Table S1: CDOCKER interaction energy for piceatannol, gnetol, resveratrol, rhapontigenin, and isorhapontigenin docked in AChE and Aβ peptide (1–42). Figure S1: Effects of different concentrations (1, 2, 5, and 10 μM) of hydroxylated stilbenes (PIC, GNT, RSV, RHA, and isoRHA) on cytotoxicities of SH-SY5Y cells. The MTT was used to evaluate cell viability, and the absorbance at 570 nm was determined. The DMSO used instead of the sample solution was blank (recognized as 100%), and each treatment was expressed as relative cell viability (%). Figure S2: Validation of the docking protocol using donepezil. The co-crystallized ligand was re-docked to confirm the docking protocol of AChE. The co-crystallized ligand (light blue) and the docked ligand (cyan) displayed similar poses. The RMSD values of the re-docking results in AChE is 1.6494 Å. The catalytic anionic site (CAS) and the peripheral anionic site (PAS) of AChE.
