# Supplementary material for: Inhibition of Acetylcholinesterase and Amyloid-β Aggregation by Piceatannol and Analogs: Assessing In Vitro and In Vivo Impact on a Murine Model of Scopolamine-Induced Memory Impairment
Source: Antioxidants (Basel). 2023 Jun 29;12(7):1362. doi: 10.3390/antiox12071362 (PMC10376691; doi:10.3390/antiox12071362)
Supplement: Supplementary file 1 [file antioxidants-12-01362-s001.zip › antioxidants-2453099-supplementary.pdf]

Inhibition of Acetylcholinesterase and Amyloid-β Aggregation by Piceatannol and Analogs: Assessing In Vitro and In Vivo Impact on a Murine Model of Scopolamine-Induced Memory Impairment

Yi-Yan Sie[1,†], Liang-Chieh Chen[2,3,†], Cai-Jhen Li[4], Yu-Hsiang Yuan[4], Sheng-Hung Hsiao[4], Mei-Hsien Lee[4], Ching-Chiung Wang[5,6,*] and Wen-Chi Hou[4,*]

[1] Ph.D. Program in Clinical Drug Development of Herbal Medicine, College of Pharmacy, Taipei Medical University, Taipei, Taiwan

[2] Department of Pharmacology and Pharmaceutical Sciences, School of Pharmacy, University of Southern California, Los Angeles, CA, United States

[3] Graduate Institute of Cancer Biology and Drug Discovery, College of Medical Science and Technology, Taipei Medical University, Taipei, Taiwan

[4] Graduate Institute of Pharmacognosy, Taipei Medical University, Taipei 110, Taiwan

[5] School of Pharmacy, Taipei Medical University, Taipei 110, Taiwan

[6] Traditional Herbal Medicine Research Center, Taipei Medical University Hospital, Taipei, Taiwan

* Correspondence: crystal@tmu.edu.tw (C.-C.W.); wchou@tmu.edu.tw (W.-C.H.)

† These two authors have contributed equally to this work.

Supplementary materials

Table S1. CDOCKER interaction energy for piceatannol, gnetol, resveratrol, rhapontigenin, and isorhapontigenin docked in AChE and Aβ peptide (1-42)

Compound	AChE	Aβ peptide (1-42)
	CDOCKER Interaction Energy (kcal/mol)	CDOCKER Interaction Energy (kcal/mol)
**Piceatannol**	-36.3392	-19.3083
**Gnetol**	-34.6363	-13.7451
**Resveratrol**	-31.4709	-17.0259
**Rhapontigenin**	-34.5302	-14.7856
**Isorhapontigenin**	-35.1776	-14.2737

Figure S1

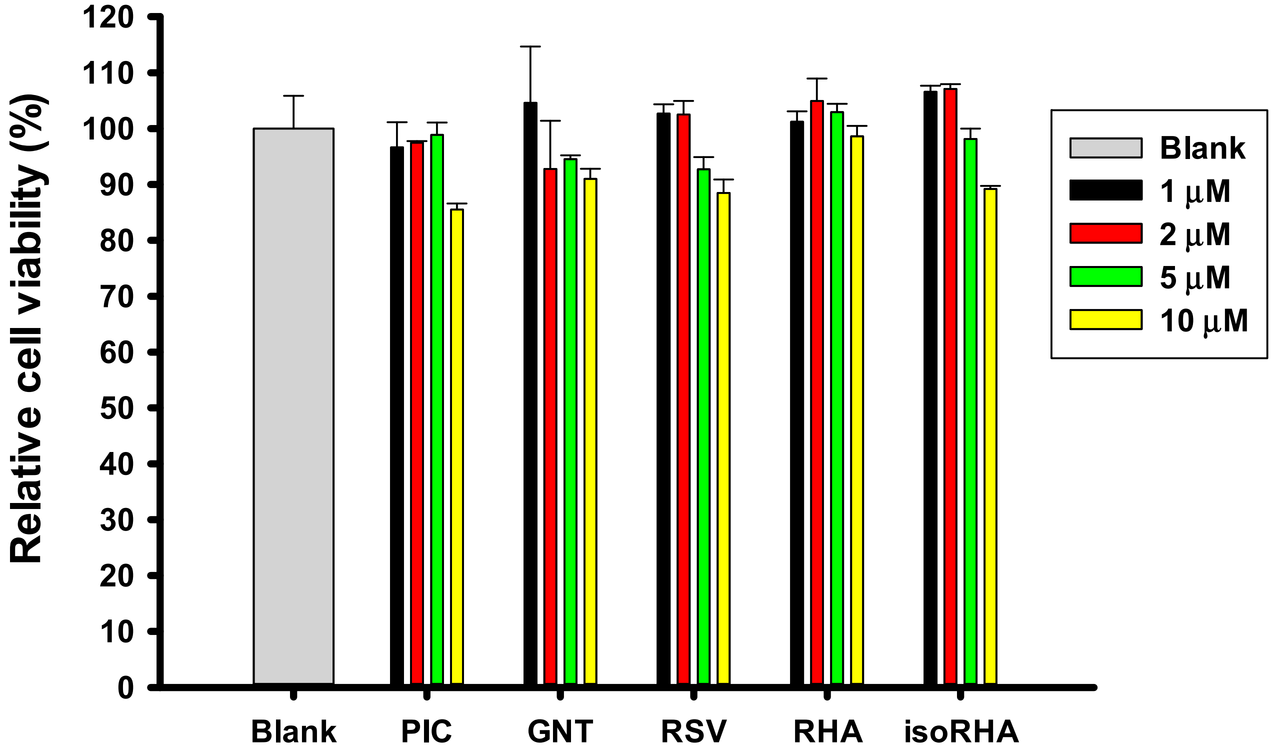

Figure S1. Effects of different concentrations (1, 2, 5, and 10 μM) of hydroxylated stilbenes (PIC, GNT, RSV, RHA, and isoRHA) on cytotoxicities of SH-SY5Y cells. The MTT was used to evaluate cell viability, and the absorbance at 570nm was determined. The DMSO used instead of sample solution was the blank (recognized as 100%), and each treatment was expressed as relative cell viability (%).

Figure S2

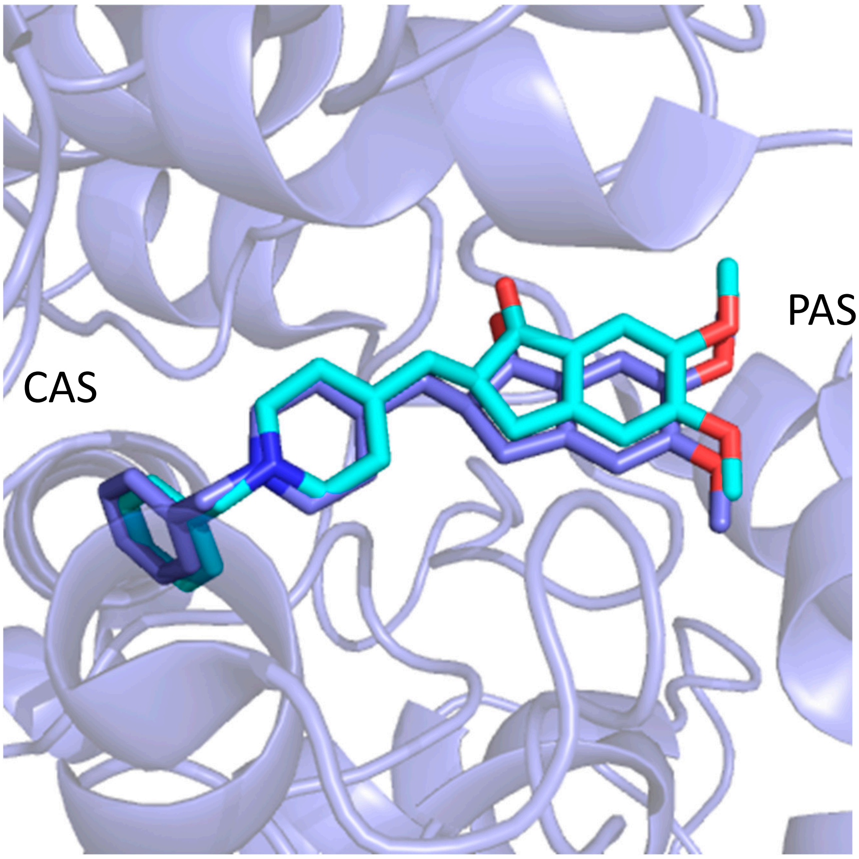

Figure S2. Validation of the docking protocol by using donepezil. The co-crystallized ligand was re-docked to confirm the docking protocol of AChE. The co-crystallized ligand (light blue) and the docked ligand (cyan) displayed similar poses. The RMSD values of the re-docking results in AChE is 1.6494 Å. The catalytic anionic site (CAS) and the peripheral anionic site (PAS) of AChE.
